# DDBJ update: streamlining submission and access of human data

**DOI:** 10.1093/nar/gkaa982

**Published:** 2020-11-06

**Authors:** Asami Fukuda, Yuichi Kodama, Jun Mashima, Takatomo Fujisawa, Osamu Ogasawara

**Affiliations:** Bioinformation and DDBJ Center, National Institute of Genetics, Mishima, Shizuoka 411–8540, Japan; Bioinformation and DDBJ Center, National Institute of Genetics, Mishima, Shizuoka 411–8540, Japan; Bioinformation and DDBJ Center, National Institute of Genetics, Mishima, Shizuoka 411–8540, Japan; Bioinformation and DDBJ Center, National Institute of Genetics, Mishima, Shizuoka 411–8540, Japan; Bioinformation and DDBJ Center, National Institute of Genetics, Mishima, Shizuoka 411–8540, Japan

## Abstract

The Bioinformation and DDBJ Center (DDBJ Center, https://www.ddbj.nig.ac.jp) provides databases that capture, preserve and disseminate diverse biological data to support research in the life sciences. This center collects nucleotide sequences with annotations, raw sequencing data, and alignment information from high-throughput sequencing platforms, and study and sample information, in collaboration with the National Center for Biotechnology Information (NCBI) and the European Bioinformatics Institute (EBI). This collaborative framework is known as the International Nucleotide Sequence Database Collaboration (INSDC). In collaboration with the National Bioscience Database Center (NBDC), the DDBJ Center also provides a controlled-access database, the Japanese Genotype–phenotype Archive (JGA), which archives and distributes human genotype and phenotype data, requiring authorized access. The NBDC formulates guidelines and policies for sharing human data and reviews data submission and use applications. To streamline all of the processes at NBDC and JGA, we have integrated the two systems by introducing a unified login platform with a group structure in September 2020. In addition to the public databases, the DDBJ Center provides a computer resource, the NIG supercomputer, for domestic researchers to analyze large-scale genomic data. This report describes updates to the services of the DDBJ Center, focusing on the NBDC and JGA system enhancements.

## INTRODUCTION

The DNA Data Bank of Japan (DDBJ) (1) is a public nucleotide sequence database established at the Bioinformation and DDBJ Center (DDBJ Center, https://www.ddbj.nig.ac.jp) of the National Institute of Genetics (NIG). Since 1987, the DDBJ has been collecting nucleotide sequences with annotations in collaboration with GenBank (2) at the National Center for Biotechnology Information (NCBI) and the European Nucleotide Archive (ENA) (3) at the European Bioinformatics Institute (EBI). This collaborative framework is known as the International Nucleotide Sequence Database Collaboration (INSDC) (4).

Within the INSDC framework, the DDBJ Center also provides the DDBJ Sequence Read Archive (DRA) for raw sequencing data and alignment information from high-throughput sequencing platforms (5), BioProject for study information, and BioSample for sample information (1,6). This comprehensive resource of nucleotide sequences enriched with contextual information relating to studies and samples complies with the INSDC policy that guarantees free and unrestricted access to all public data (7). In addition to these INSDC databases, the DDBJ Center has accepted data from functional genomics experiments (e.g., gene expression and epigenetics) in the Genomic Expression Archive (GEA) (8) as the Gene Expression Omnibus at NCBI (9) and the ArrayExpress at EBI (10).

For controlled-access data, the DDBJ Center services the Japanese Genotype–phenotype Archive (JGA) (1,11). The JGA archives and distributes human genotype and phenotype data, as the database of Genotypes and Phenotypes (dbGaP) at NCBI (12) and the European Genome–phenome Archive (EGA) at EBI (13) do. The guidelines and policies for accessing human data (https://humandbs.biosciencedbc.jp/en/guidelines) were codified by the National Bioscience Database Center (NBDC, https://biosciencedbc.jp/en/) at the Japan Science and Technology Agency, and its Data Access Committee (DAC) has been reviewing applications for data submission and JGA access.

To simplify the process of application to NBDC and JGA access, we have introduced a unified login platform in September 2020. In this new system, users can submit applications to NBDC, and access data in JGA through the same accounts. The new platform supports a group account system, which enables the flexible management of data submitters and users.

To cope with the increasing submissions, we are working to automate submission processes while maintaining quality of data. As part of such effort, quality checks were introduced to the DDBJ Fast Annotation and Submission Tool (DFAST, https://dfast.nig.ac.jp), which is a prokaryotic genome annotation and submission pipeline producing submission-ready DDBJ annotation files (14).

To improve search functionalities of vast amounts of metadata, we introduced Resource Description Framework (RDF). In collaboration with the Database Center for Life Science (DBCLS), structured queries to the BioSample metadata were realised by mapping the metadata to ontology terms in RDF.

Besides the public databases for biological sciences, the DDBJ Center provides a computer resource, the NIG supercomputer, for domestic researchers to analyze large-scale genomic data. To enhance capabilities of large-scale data store and analysis, storage and calculation nodes have been added to the supercomputer.

In this article, we report updates to the services of the DDBJ Center, focusing on the NBDC and JGA system enhancements. All resources are available at https://www.ddbj.nig.ac.jp, and the data are downloadable at ftp://ftp.ddbj.nig.ac.jp.

## DDBJ ARCHIVAL DATABASES

### Data contents: unrestricted- and controlled-access databases

In 2019, the DDBJ accepted 6688 submissions of annotated nucleotide sequences, 74.5% of which were contributed by Japanese research groups. The DDBJ has periodically released all public DDBJ/ENA/GenBank nucleotide sequence data in the flat file format. The latest periodical release of June 2020 contains 2 414 499 799 sequences and 253 936 453 958 bp, and the DDBJ contributed 3.52% of the sequences and 2.48% of the base pairs.

In 2019, the DRA accepted 38 716 runs of high-throughput sequencing data. As of August 2020, the DRA distributed 8.7 PB of sequencing data in the SRA (7.5 PB) and FASTQ (1.2 PB) formats. The DRA has resumed NCBI/EBI SRA data mirroring after the expansion of storage space last year, and mirrored most of the data in July 2020. In 2019, the GEA accepted 61 submissions of data from functional genomics experiments. As of August 2020, the GEA provided 31 experiments from its ftp site (ftp://ftp.ddbj.nig.ac.jp/ddbj_database/gea).

The JGA is a controlled-access database for genotypes and phenotypes of human individuals (11). In 2019, the JGA accepted 49 studies and 24,422 samples submitted by Japanese research groups. As of August 2020, the JGA distributed 124 studies, 248 240 samples, and 256 TB of human data. Summaries of these studies are available to the public on both the DDBJ Search (https://ddbj.nig.ac.jp/search) and the NBDC (https://humandbs.biosciencedbc.jp/en/data-use/all-researches) websites. As one of the Global Alliance for Genomics and Health (GA4GH, https://www.ga4gh.org/) Driver Projects, the GEnome Medical alliance Japan (GEM Japan) has analyzed whole-genome sequences from 7609 Japanese individuals and publicly released aggregated variant and frequency datasets at the variant data portal ‘TogoVar’ (https://togovar.biosciencedbc.jp) of NBDC (https://www.amed.go.jp/en/news/release_20200727.html). Some of the underlying whole-genome sequencing and alignment information is available at the JGA under study accession numbers JGAS000109, JGAS000114, JGAS000151, JGAS000155 and JGAS000239. To use controlled-access data, data use applications should be submitted to NBDC (https://humandbs.biosciencedbc.jp/en/data-use). The JGAS000239 study summary is publicly available at the JGA website, while the whole-genome sequence (WGS) data of 4566 Japanese individuals are accessible at the submitter's Tohoku Medical Megabank Organization (ToMMo) supercomputer. An overview of the statistics is available on our website (https://www.ddbj.nig.ac.jp/statistics-e.html).

### The enhanced systems for NBDC and JGA

**Figure 1.**
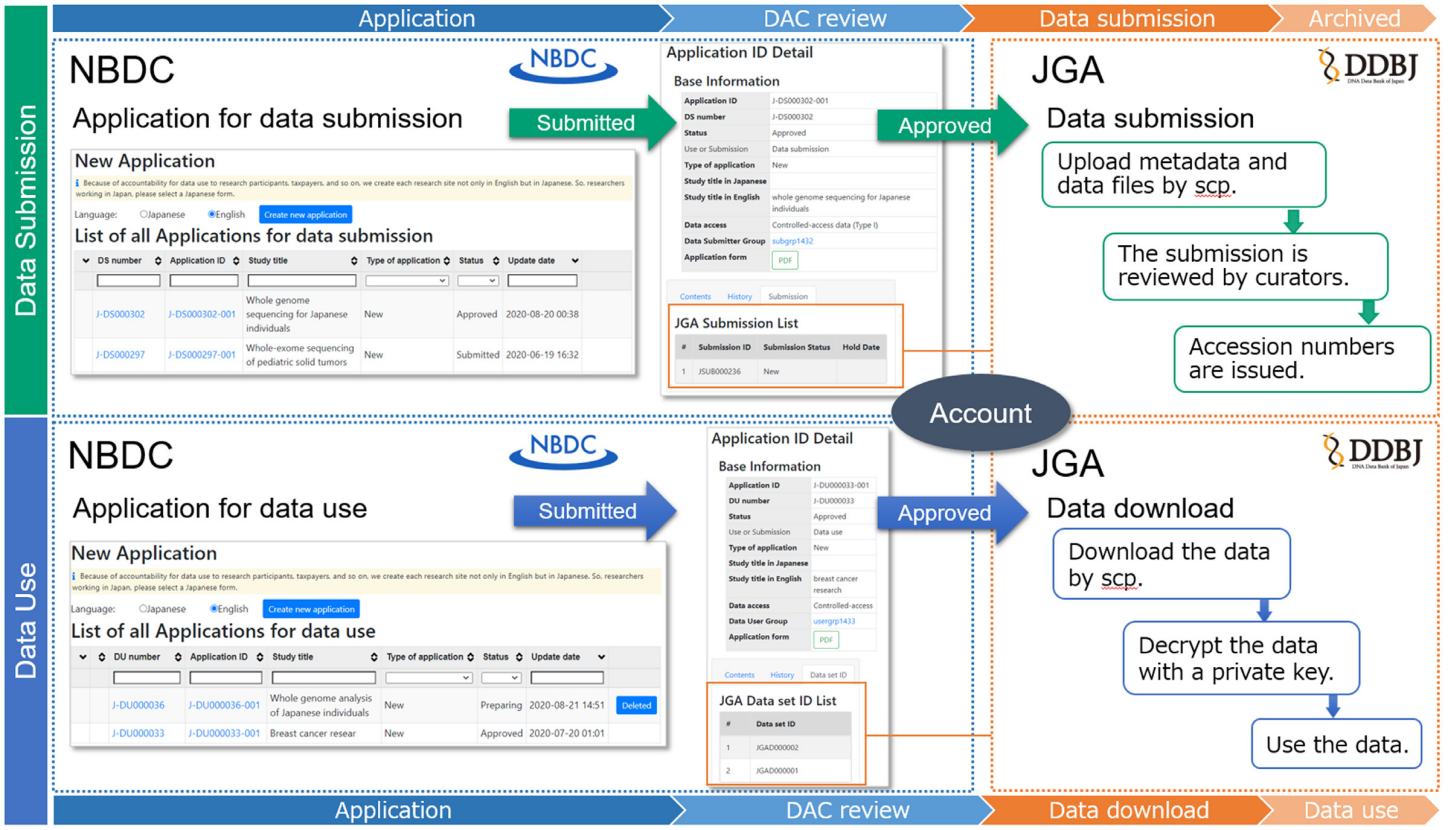
The processes of data submission and data use at NBDC and JGA. For data submission, users submit applications to NBDC. After the applications have been approved by the DAC of NBDC, users can upload data to JGA. For data use, users submit applications to NBDC. After the applications have been approved by the DAC of NBDC, users can download data from JGA. Users can carry out all processes in the common login platform.

As human genomes are produced by high-throughput sequencing platforms, data submissions/downloads in JGA and applications to NBDC increase. Previously, NBDC accepted applications through emails only. Revision and update of the applications were manually performed. In September 2020, to minimize the manual processes, the NBDC released a web-based system connected with JGA by a unified login platform. In this new system, users can submit applications to NBDC and also upload/download data files in JGA through the same accounts (Figure 1). In the web-based system, users can view applications and linked submissions/downloads with their respective statuses (e.g. application status is ‘Approved’ and linked data submission status is ‘Data Submitted’), so that they can become aware of any progress that has occurred, and revise and update the applications.

**Figure 2.**
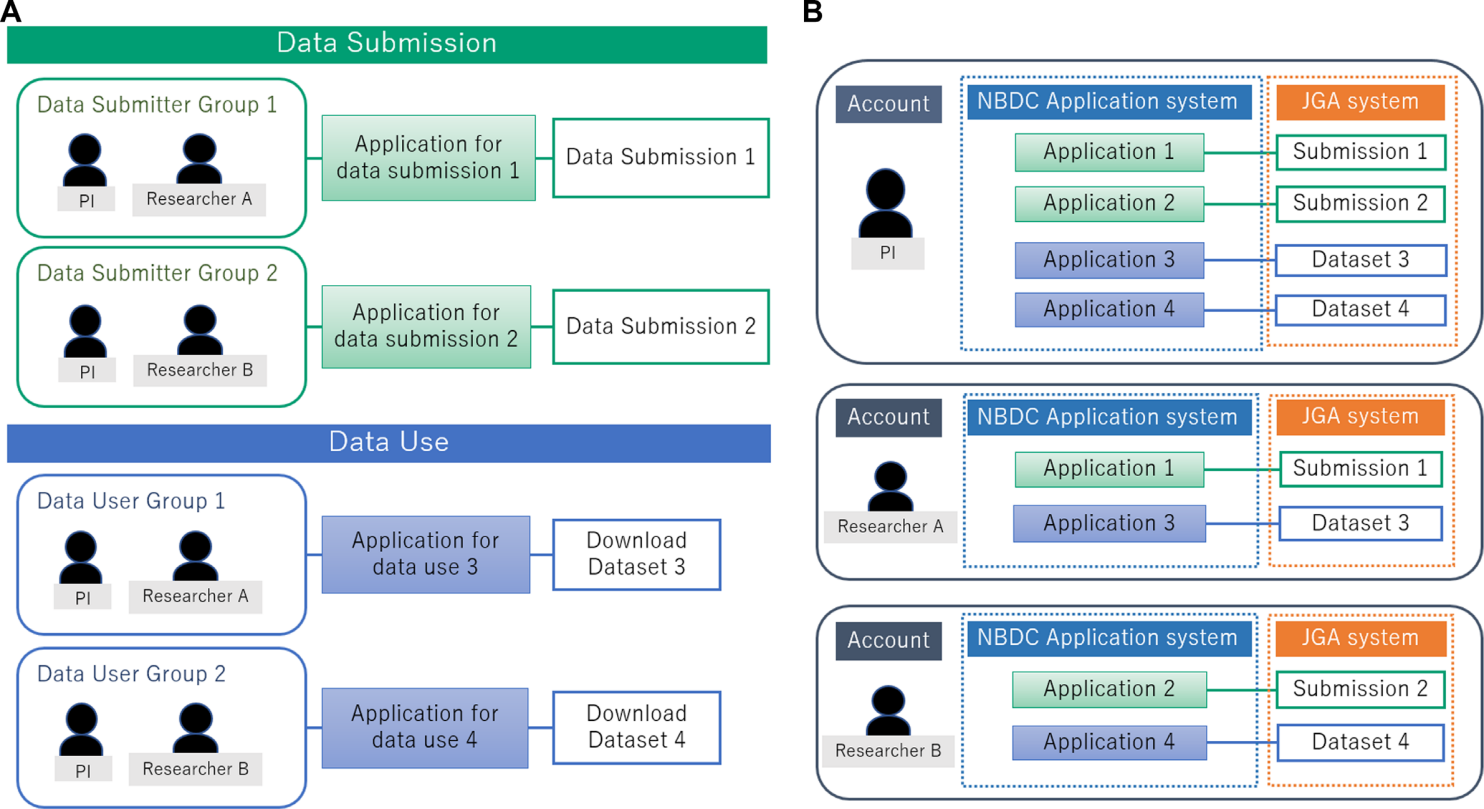
The groups of data submitters and users. (**A**) The application to NBDC and data submission to JGA are managed by the data submitter group. Similarly, the application to NBDC and data download from JGA are managed by the data user group. The principal investigator (PI) and researchers are able to manage the data without sharing their accounts. The PI can manage data from multiple projects by creating groups with different project members (for example, one with the researcher A and the other with the researcher B). (**B**) The researchers A and B can access only the data associated as group members, while the PI can access all the data.

Now the applications, data submissions, and data downloads are managed through the unified group account system. For example, an application to NBDC and a data submission at JGA are linked to a submitter group consisting of a principal investigator (PI) and fellow researchers. Through this group, the PI can check documents, metadata, and sequencing data submitted by the researchers without sharing the accounts. The PI can also manage data submissions and downloads of multiple projects by creating groups with different project members (Figure 2).

Approved datasets can be downloaded from the JGA server by the standard Secure Copy Protocol (scp). To improve security, an encryption/decryption step has been added to the data use process. The datasets at JGA are encrypted by a public key registered by a user during the data use application process at NBDC. After downloading the datasets, the user needs to decrypt the datasets by using a private key paired with the registered public key. Because the datasets are encrypted by the standard Advanced Encryption Standard (AES), decryption performance can be accelerated by modern Intel and AMD CPUs. Downloaded datasets can be stored in encrypted form while not in use, for security.

The JGA datasets, especially WGS datasets, have increased in size. For example, JGAS000114 containing WGS data of 1026 Japanese individuals is 75 TB. The large size of the JGA sequencing datasets makes it difficult for users to prepare their own secure computer resources with sufficient storage and computing power and to transfer large data. To solve these issues, the DDBJ Center has provided a secured NIG supercomputer environment for analyzing personal genome data. Users are allowed to analyze approved JGA datasets in the secured NIG supercomputer in addition to their own servers. Because the secured NIG supercomputer is connected with the JGA server through a high-speed network, users can smoothly download JGA datasets and analyze their own personal genomic data in the same environment.

## DDBJ SYSTEM UPDATE

### Services for submitting biological data

As sequencing technologies are changing and submissions are increasing, our submission processes are transitioning from manual curation to an automatic format. We are working to automate submissions of prokaryotic genomes by using the DFAST. In July 2020, a new feature, ‘DFAST Quality Control’ (DQC), was introduced to DFAST to improve the quality of genome submissions. DQC checks the taxonomic assignment of the genome by using average nucleotide identity (ANI), and estimates contamination and completeness scores. More than 90% of prokaryotic genomes are routinely submitted to DDBJ through DFAST.

### Services for retrieving and analyzing biological data

The DDBJ Center provides services for searching, retrieving, and analyzing nucleotide sequences and taxonomies. We provide services for downloading public data in our archival databases and those in other mirrored databases at ftp://ftp.ddbj.nig.ac.jp. The DDBJ Center is developing an integrated search service, DDBJ Search (https://ddbj.nig.ac.jp/search), which aims to index metadata of all databases of the DDBJ Center. The DDBJ Search indexes metadata in the json file format by using Elasticsearch (https://www.elastic.co/elasticsearch). In September 2020, we released the DDBJ Search indexing JGA public metadata as a first version. The DDBJ Search will be expanded to include metadata of the other databases.

The BioSample database allows submitters to add their own original attributes to flexibly describe samples, but these attributes tend to be less standardized. Thus, standardization of the BioSample attributes by ontology terms is necessary to enable queries to be performed in a more structured way. In collaboration with the DBCLS, we provide the BioSample records mapped to ontology terms in the RDF format at the ftp site (ftp://ftp.ddbj.nig.ac.jp/rdf/biosample/).

### The NIG supercomputer

The NIG supercomputer serves as a large-scale computational resource for Japanese researchers. To accommodate the growing amount of sequencing data, the supercomputer was replaced in early 2019. The new system is equipped with 27.9 PB of sequencing data archiving storage (12.9 PB disk and 15 PB tape), large-scale parallel distributed file systems (13.8 PB in total), and 1.1 PFLOPS calculation nodes and graphics processing units (GPUs). The secured supercomputer environment for personal genome analysis is composed of 46 calculation nodes and 5 PB of the parallel distributed file systems. The large datasets available at our ftp site are also directly accessible in the supercomputer file system. In late 2020, 3.6 PB of the parallel distributed file systems will be added to enhance the storage capacity. To ensure reproducibility of analysis, pre-installed bioinformatics tools are provided in the Docker/Singularity containers and users can build, run, and deploy the containers in the supercomputer. In collaboration with DBCLS, the Sapporo workflow execution web service, which is compatible with the GA4GH Workflow Execution Service (WES) standard (https://github.com/ga4gh/workflow-execution-service-schemas), has been implemented. The NIG supercomputer is connected with Amazon Web Services (AWS) so that users can choose an appropriate analytical environment.

## FUTURE DIRECTION

Owing to the evolution of technologies measuring biological molecules, rapidly growing amounts of diverse and complex data are produced in the life sciences. It is important for data-intensive science that data are findable, accessible, interoperable, and reusable (FAIR), both for machines and for humans (15). To archive and provide comprehensive biological datasets, the DDBJ Center has established the following databases: DRA (2008), BioProject (2011), JGA (2013), BioSample (2014) and GEA (2018). Furthermore, the DDBJ Center will launch the MetaboBank for metabolomics data in October 2020 and the Japan Variation Archive (JVar) for human variation data in early 2021. We use the Semantic Web technologies for data integration and definition of rules. The BioSample and DDBJ records are available in RDF formats at our ftp site (ftp://ftp.ddbj.nig.ac.jp/rdf) and at the NBDC RDF portal (https://integbio.jp/rdf). The BioSample packages, collections of mandatory and optional attributes for each sample type, are defined in the web ontology language OWL and will be used by the BioSample submission service. A unified OWL is under development to represent validation rules of all data types at the DDBJ Center's databases.
